# Adaptation of Work Values Instrument in Indonesian Final Year University Students

**DOI:** 10.3389/fpsyg.2022.858688

**Published:** 2022-05-12

**Authors:** Rezki Ashriyana Sulistiobudi, Harlin Nikodemus Hutabarat

**Affiliations:** Department of Industrial and Organizational Psychology, Faculty of Psychology at Universitas Padjadjaran, Bandung, Indonesia

**Keywords:** work values, instrument adaptation, validity, reliability, confirmatory factor analysis

## Abstract

**Background:**

One of the preferences working in the Generation Z is based on their motivational work values. The relevance of job choices with the work values will contribute to student career planning. The work value instrument among generations is one of the popular instruments used to measure final year students' work value, yet few studies of the psychometric properties of non-English language versions of this instrument. This study's objectives were to adapt a questionnaire of work value in Indonesian final year university students.

**Methods:**

The number of participants in this study was 316 students in Indonesia, comprised of final year students from various majors who were selected by quota sampling. The instrument consisted of 5 dimensions of value, including leisure, extrinsic rewards, intrinsic rewards, altruistic rewards, and social rewards. The reliability analysis was performed using McDonald's Omega, the evidence of validity was obtained from test content, internal structure through confirmatory factor analysis (CFA), and evidence-based in relation to other variable has conducted the correlation between work value and career development learning using the Pearson's correlation coefficient.

**Results:**

The results showed that the work values instrument had good psychometric properties, including good reliability, good content validity, and internal structure. In CFA, the two-factor structure showed satisfactory model fit. Moreover, the correlation of work value with career development learning builds stronger validity evidence on this instrument.

**Conclusion:**

The adapted instrument can be used practically to identify work value preferences of final year students to help them choose a work preference and setup the career planning before graduating. The result could be of interest for the researcher in work value, motivational work, and career areas in higher education. To the best of our knowledge, there have been no reports about the adaptation of work value instruments in Indonesian final year university students.

## Introduction

Students' college years are critical to explore working interests and prepare for their first jobs. During college years, students learn about themselves, including their needs, motives, and working preferences for their future careers. Work values refer to what people desire to do or to have at work (De Cooman and Dries, 2012; Ros et al., 2015) and needs and motives refer to individuals' preferences for work. Work values act as “secondary drivers of action that are determined by needs as well as socialization, cognition, and experience” (Kooij et al., 2011). In simple terms, work values indicate what is important or desirable to individuals in their working lives (Kuron et al., 2015). Work values have a high degree of stability and wide impact on career plans and decisions and will result in various aspects at work, including individual and organizational outcomes such as work motivation, work attitudes, career choices, task preferences, decision-making, resistance to organizational change, and overall managerial success (Schleicher et al., 2011).

Related to career, choosing the suitable type of work may begin with identifying of student's own work values. Since completion of higher education will lead young adults to enter the labor market immediately and work full time (Santrock, 2019), the current employee selection process is quite challenging, especially for college fresh graduates. Understanding one's characteristics and identity are essential for graduates to be more proactive in pursuing their career goals (Kadiyono et al., 2020). By the time, they have entered the job sector that suits their values and they will be able to achieve better performance (Lin et al., 2015). Based on studies, choosing a careerrelevant to the meaningful values possessed will impact one's ability to perform the work, which, in turn, affects one's wellbeing. When their work values are aligned with the organization's values, they will be happier, be more motivated, more satisfied, and more committed (Hikspoors, 2011; Rani and Samuel, 2016). To this end, it becomes critically important to understand students' work values as they enter the workforce and develop their careers.

The development of work values has shown its significant role in the scope of the workforce and professional environment. Work value has been an interesting topic since Weber's (1958) s study on Protestant Work Ethic (PWE) (Parry and Urwin, 2011). However, PWE's prominence has declined and has been replaced by a wider range of work values suited to the modern workplace. According to Dose (1997), the study of work values measurement continues to grow from the creation of Super's Work Values Inventory (WVI) in the 1950s and Pryor's Work Aspect Preference Scale (WAPS) in 1981 (Pryor, 1981) that are well-known representatives of the former end of the continuum. The latter end of the continuum includes values such as asceticism (Mirels and Garrett, 1971) or humanistic beliefs [Belief about Work Questionnaire (Buchholz, 1978)]. Other scales from Dawis and Lofquist's Minnesota Importance Questionnaire (MIQ) in 1984, the integration of WVI, WAPS, and MIQ by Macnab and Fitzsimmons in 1987, and the redefinition of work values by Dose in 1997.

Study has found that different generations have different characters, personalities, needs, and values (Twenge et al., 2010; De Cooman and Dries, 2012; Tolbize, 2014). Therefore, the latest development of work value measurement is taking into account generativity that can reflect a broad range of different work values in different generations (Twenge et al., 2010; Krumm et al., 2013; Singh et al., 2020; Song et al., 2020). As of, the instrument can be used for the latest generation (Generation Z), which is the final year students in higher education. Thus, far, many of the studies addressing this importance of understanding the work value in final year students and graduates (De Cooman and Dries, 2012; Shujaat, 2014; Kuron et al., 2015; Ros et al., 2015; Chi et al., 2019; Doo and Park, 2019; Hampton and Welsh, 2019; Neyt et al., 2019). However, the instrument used was not specific for students in higher education. It becomes important since we agreed that the student's character as Generation Z is different from the older generation.

The most comprehensive study suggest to consider four broader work value domains: (1) Intrinsic, (2) Extrinsic, (3) Social/Relational, and (4) Prestige. The four higher-order domains of work values may have the potential to summarize most of the needs and values that individuals seek and try to satisfy through working (Jin and Rounds, 2012; Abessolo et al., 2017). We considered that those four work value domains similar to the scale from Twenge et al. (2010), which their work has become the most widely cited generational work values perspective to date. The instrument successfully captured the motivational value differences of each generation. According to Twenge et al. (2010), there are 5 (five) categories of work values:

**1. Leisure**

Leisure is the value when students are more interested in jobs that can accommodate their family and personal life. Based on popular thinking, they seek jobs with telecommunications options and flexibility at work to take care of children and family and travel or spend more time with friends. Fulfilling those needs will result in a better career (Kelly et al., 2020).

**2. Extrinsic rewards**

Extrinsic rewards such as salary, property, and prestige are the main factors that motivate people to work. Given current trends in the economic situation, employees in the current generation may increasingly emphasize jobs that provide extrinsic rewards. The extrinsic reward will result in employees' high performance and wellbeing (Merriman, 2017).

**3. Intrinsic rewards**

Intrinsic rewards refer to work motivation, which requires an increased ability in the related field. Intrinsic rewards in work values do not refer to the desire to receive material or extrinsic rewards. Intrinsic rewards include challenging jobs, enhancing skills, and allowing employees to develop (Porfeli and Mortimer, 2010), providing variety and responsibility, offering challenges, allowing employees to acknowledge their outcomes, and significantly impacting others. Employee recruitment, selection, and training are currently managed to emphasize employees' potential and career growth.

**4. Altruistic rewards**

Altruistic rewards include motivation to help others and society through work. Altruism is one's willingness to help others without considering their personal interests (Adhiatma and Fachrunnisa, 2021). Some companies establish community programs to attract younger employees to introduce various programs that allow employees to work as volunteers while working at the company.

**5. Social rewards**

In social relations, a person must be in constant contact either formally or informally for a long time (Jo and Ellingson, 2019). Social rewards are the need to relate, connect with social groups in various occupations. Nowadays, social networking sites can create an impression of the need to connect and document social relationships with others to increase the need to connect with social activities.

The instrument has been validated with respondents from high schools students across the United States Model fit indices of the instrument supported the appropriateness of the five-factor solution based on the remaining 19 items. The reliability of the five factors appeared satisfying. This scale in the English language was widely used around the world. To the best of our knowledge, the scale is still not available in the Indonesian context. We hope that the availability of a valid tool to measure work value in final year students will foster the study and practically help the student to plan their career based on the work value. This study facilitates access to a much-needed instrument measuring work motivational value who wish to conduct basic and applied research in Indonesian students' context. Therefore, this study aimed to adapt the scale in the Indonesian context, specifically for final year students in higher education.

## Methods

### Participant

The participants in this study were final year undergraduate student at a public university in West Java province, Indonesia. The university was chosen for its position as one of the big ten universities in Indonesia. The sampling technique used was quota sampling, which was a form of non-probability sampling. The response rate is 89% compared with the number of selected samples. A total of 316 respondents participated in this study, representing all the faculties in the university. The study groups were structurally varied in terms of sex and faculty, as illustrated in Table 1.

**Table 1:** Respondents' demographic data.

**Category**		**Frequency (%)**
Gender	Male	31
	Female	69
Faculty cluster	Science and technology	33.54
	Health	21.52
	Social and humanities	44.94

All the participants involved in this study were confirmed their consent before fill the questionnaire. The informed consent should be include: (1) the purpose of the study, expected duration, and procedures; (2) their right to decline to participate and to withdraw from the study once participation has begun; (3) the foreseeable consequences of declining or withdrawing; (4) reasonably foreseeable factors that may be expected to influence their willingness to participate; (5) any prospective study benefits; (6) limits of confidentiality; (7) incentives for participation; and (8) whom to contact for questions about the study and study participants' rights (American Psychological Association, 2017).

### Instrument

#### Work Value Instrument

The work value instrument used in this study was developed by Twenge and colleagues (Twenge et al., 2010). The instrument consisted of 5 dimensions, including leisure, extrinsic rewards, intrinsic rewards, altruistic rewards, and social rewards. Sample of the original items for each dimension was “a job that leaves a lot of time for other things in your life” (leisure), “a job where you can learn new things and learn new skills” (intrinsic rewards), “a job that is worthwhile to society” (altruistic rewards), “a job that gives you a chance to make friends” (social rewards), and “A job where the chances for advancement and promotion are good” (extrinsic rewards). Each item used a 5-point Likert scale, ranging from 1 = very unimportant, 2 = unimportant, 3 = undecided, 4 = important, and 5 = very important (see Table 2).

**Table 2:** Work values instruments.

**Original items and translation**	
1	A job where you have more than 2 weeks' vacation
	*(Pekerjaan yang memberikan kesempatan berlibur)*
2	A job that leaves a lot of time for other things in your life
	*(Pekerjaan yang menyisakan banyak waktu untuk melakukan berbagai hal lain dalam hidup)*
3	A job with an easy pace that lets you work slowly
	*(Pekerjaan dengan irama santai, tidak terburu-buru)*
4	A job that leaves you mostly free of supervision by others
	*(Pekerjaan yang cukup bebas dari pengawasan orang lain)*
5	A job that is interesting to do
	*(Pekerjaan yang berisi tugas-tugas menarik)*
6	A job where you can learn new things, learn new skills
	*(Pekerjaan yang membuka kesempatan untuk belajar hal baru maupun keterampilan baru)*
7	A job where the skills you learn will not go out of date
	*(Pekerjaan dimana keterampilan yang dimiliki tidak akan pernah usang)*
8	A job where you can see the results of what you do
	*(Pekerjaan dimana apa yang kita kerjakan hasil akhirnya dapat dilihat)*
9	A job that uses your skills and abilities—lets you do the things you can do best
	*(Pekerjaan yang memanfaatkan keterampilan dan kemampuan diri)*
10	A job where you do not have to pretend to be a type of person that you are not
	*(Pekerjaan dimana bisa menampilkan diri apa adanya)*
11	A job where you have the chance to be creative
	*(Pekerjaan yang memberi kesempatan untuk kreatif)*
12	A job that gives you an opportunity to be directly helpful to others
	*(Pekerjaan yang memberi kesempatan untuk menolong orang lain secara langsung)*
13	A job that is worthwhile to society
	*(Pekerjaan yang memberi manfaat bagi masyarakat)*
14	A job that gives you a chance to make friends
	*(Pekerjaan yang memberi kesempatan untuk menjalin pertemanan)*
15	A job that permits contact with a lot of people
	*(Pekerjaan yang memungkinkan untuk berelasi dengan banyak orang)*
16	A job that has high status and prestige
	*(Pekerjaan dengan status sosial tinggi dan berprestise)*
17	A job that most people look up to and respect
	*(Pekerjaan yang dihormati oleh orang lain)*
18	A job that provides you with a chance to earn a good deal of money
	*(Pekerjaan yang memberikan kesempatan untuk menghasilkan banyak uang)*
19	A job where the chances for advancement and promotion are good
	*(Pekerjaan yang memberikan peluang bagus untuk pengembangan karir dan promosi)*

#### Career Development Learning

Career development learning (CDL) was developed by Pool et al. (2014). CDL is one aspect of the Employability Development Profile (EDP). The EDP was explicitly designed for developmental work with students of any higher education institution. CDL is about ensuring students are well-prepared for getting a job, but a good deal of important work in this area takes place long before graduation (Pool, 2020). CDL consists of five items, for example, “I know what I want to do when I finish my degree.” We used a 6-point Likert scale from (1) strongly disagree to (6) strongly agree.

### Procedure

This study consisted of two steps; in the first step, the instrument was adapted in Indonesian final year university students' context and in the second step, it was validated. In the adaptation stage, we translated the work value instrument from the original language into Indonesian by a professional translator. The translation was subjected to proofreading by three experts. The experts are researchers in industrial and organizational psychology and experience in translation, adaptation, and validation of scales. They compared the different translations and evaluated any semantic discrepancies (including any linguistic and conceptual issues) by consensus. The evaluation of the instrument was carried out by taking into consideration the characteristic of final year students in Indonesia. Afterward, linguist experts carried out the blind backward translation, resulting in one set of original and backtranslated versions of work values instrument and ready to validate.

We conducted two steps to evaluate the empirical validity evidence of work value instrument, i.e., evidence based on the test content, evidence based on internal structure, and evidence based on relation to other variables (Goodwin and Leech, 2003). In the evidence based on test content, we calculate the evidence quantitatively and it was carried out by 3 (three) raters (Polit et al., 2007). They were provided with the theory used, conceptual definition, operational definition, dimension, instrument grid, and items in the instrument. This proportion agreement procedure allows three experts to independently review, evaluate, and adjust the relevance of a sample of items to the dimension of content represented in an instrument, including item definitions, content, formats, and administration process. Subsequently, the team of researchers tally the proportion of cases in which the experts agree and determine the stability of their agreement. A Likert type, with four possible responses of relevancy, is used. The responses include a rating of 1 = not relevant, 2= somewhat relevant, 3= quite relevant, and 4 = very relevant. Researchers advocating the use of this approach specify that ratings of 1 and 2 are considered to be “content invalid,” whereas ratings of 3 and 4 are considered to be “content valid.” Waltz et al. (2010) indicated that “the actual Content Validity Index (CVI) is the proportion of items that received a rating of 3 or 4 by the expert reviewer.” Researchers are then collapsed four ordinal response rankings into two dichotomous categories of responses (“content invalid” and “content valid”) and the CVI becomes a two-category nominal scale (Waltz et al., 2010).

Before conducting the data collection for establishing its validity based on internal structure, a pilot study was conducted on a small sample of five university students. They were asked to indicate the clarity, understanding, and readability in each item. The instrument was then administered to the sample of participants to test the psychometric properties, i.e., assumption of normality, validity based on internal structure, validity based on relation to other variables, and reliability.

### Data Analysis

Before conducting the main statistical analysis, several basic assumptions should be made first. We used the Mahalanobis distance as a recommended approach for multivariate outlier detection (Finch, 2012). We then compared the Mahalanobis distance with the center of the data and excluded 6 data. Afterward, we checked the assumption of normality using the scores of kurtosis and skewness outright with the missing data checking. The Shapiro–Wilk value is 0.974 (*p* > 0.05) and it tells us that the distribution of the sample is not significantly different from a normal distribution. The value is considered acceptable for normal distribution and no missing data are found. The skewness score is −0.615, which indicates a buildup of high scores and the Kurtosis score is 1.257, which means a heavy-tailed distribution (Field, 2018).

We calculated the evidence based on test content using the Content Validity Index (CVI). We applied the universal agreement among experts, defining the Scale Level-Content Validity Index (S-CVI) as the proportion of items on an instrument that achieved a rating of 3 or 4 by all the content experts. We calculate the Item-Content Validity Index (I-CVI) for each item on the scale and then calculate the average I-CVI across items. The validity evidence based on internal structure examines the extent to which internal components of a test match the defined construct and it is estimated by confirmatory factor analysis (CFA). The CFA is a type of structural equation modeling related to the measurement model, including the relationship between the observed and latent variables (Brown, 2015). The method is an internal structure evidence collection for item discrimination (Goodwin and Leech, 2003). The analysis used the maximum-likelihood estimation method. Evidence based on relation to other variables was measured using a correlation between work value and each dimension and the career development learning. We calculated the Pearson's bivariate correlation index. We also calculated the basic psychometric of the dimensions obtained (mean and SD). The reliability of the work value instrument was estimated using McDonald's Omega (ω) instead of the Cronbach's alpha. The advantage of using Omega is that reliability is not biased, despite how low, high, or unequal the factor loadings are and the 95%CI accompanies coefficient ω to give highly probable values of reliability in the population (Goodboy and Martin, 2020; Hayes and Coutts, 2020). All the calculations were done by SPSS version 23.0 for Windows and JASP version 0.16.1.

## Results and Discussion

### Results

#### Evidence Based on Test Content

The content validity of the work values instrument is good. The I-CVI obtained was 1.00, the S-CVI/UA obtained was 1.00, and the S-CVI/Ave obtained was 1.00 (see Table 3). Polit and Beck (2006) indicated that items with an I-CVI of 0.78 or higher for at least three experts could be considered evidence of good content validity.

**Table 3:** Item rated for evidence based on test content validity.

**Item**	**Expert 1**	**Expert 2**	**Expert 3**	**Expert agreement**	**Item CVI**
1	√	√	√	4	1.0
2	√	√	√	4	1.0
3	√	√	√	4	1.0
4	√	√	√	4	1.0
5	√	√	√	4	1.0
6	√	√	√	4	1.0
7	√	√	√	3	1.0
8	√	√	√	3	1.0
9	√	√	√	3	1.0
10	√	√	√	4	1.0
11	√	√	√	4	1.0
12	√	√	√	4	1.0
13	√	√	√	4	1.0
14	√	√	√	4	1.0
15	√	√	√	4	1.0
16	√	√	√	4	1.0
17	√	√	√	4	1.0
18	√	√	√	4	1.0
19	√	√	√	4	1.0
Proportion relevant	1.0	1.0	1.0	Average I-CVI	= 1.0

#### Evidence Based on Internal Structure

**Figure 1:**
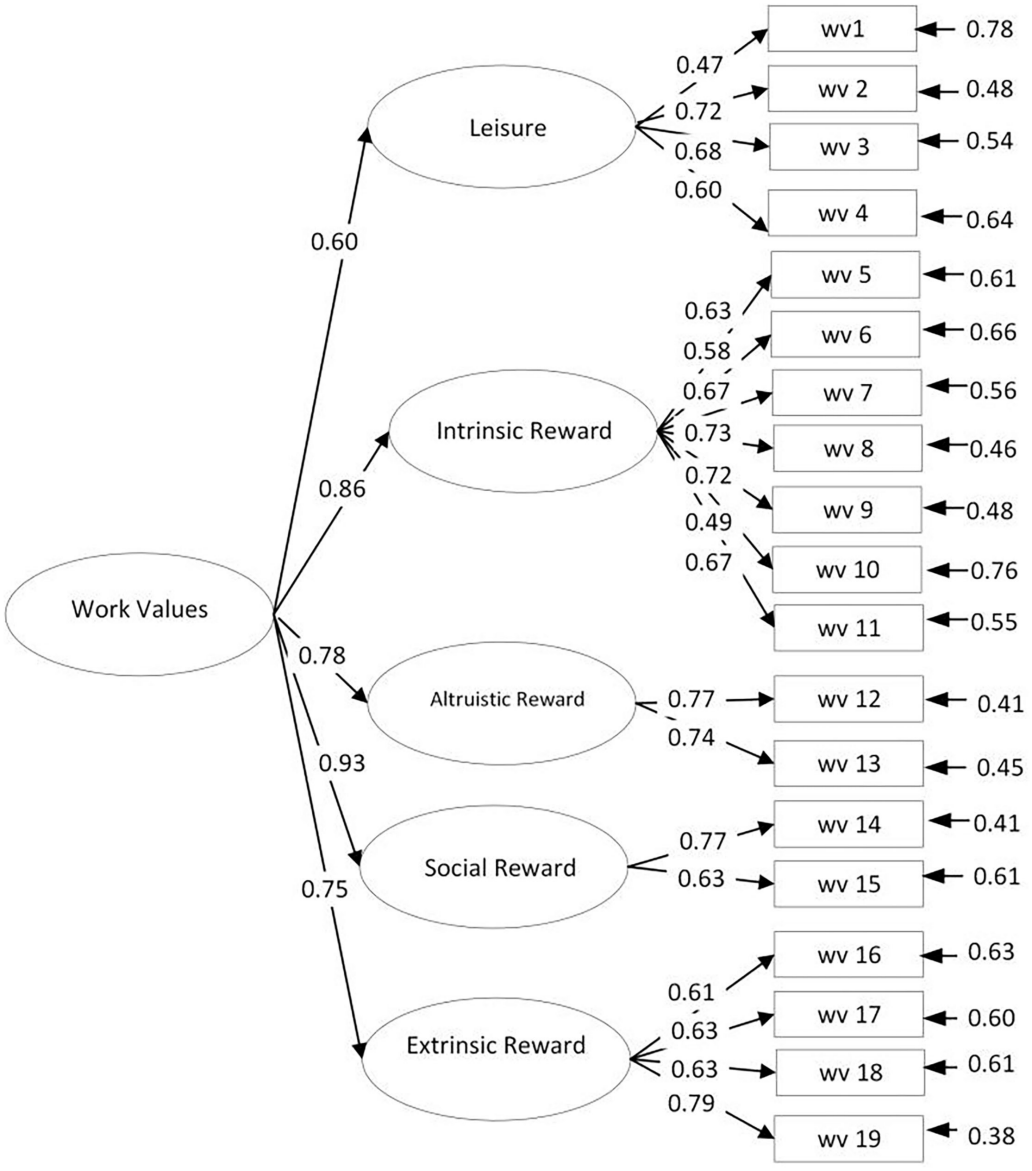
Factor analysis of work value.

Internal structure on work value was analyzed using confirmatory factor analysis. The indicators of fit indices include (1) robust comparative fit index (CFI), (2) Relative Noncentrality Index (RNI), (3) robust standardized root-mean-square residual (SRMR), and (4) robust root mean square of approximation (RMSEA). The Global Fit Indices of model 1 were χ^2^ = 663.231; d.f. = 152; *p* = < 0.001, while the Global Fit Indices of model 2 were χ^2^ = 402.303; d.f. = 147; *p* = < 0.001. We applied the threshold recommended by Hu and Bentler (1999). Model 1 shows CFI (0.905) closed to the cutoff value, RNI (0.905) is closed to the cutoff value, and SRMR (0.106) and RMSEA (0.103) exceed the cutoff value. Model 2 (second-order factor) shows better fit indexes than model 1 (see Table 4). Model 2 shows CFI (0.953) and RNI (0.953) closed to the cutoff value and SRMR (0.086) and RMSEA (0.074) also closed to the cutoff value. We can conclude that the fit values of the second-order model are best appropriate (see Figure 1). The model diagram showed that the loading factor of the 19 items in this study was >0.40, meaning that all the items are good as an observed variable (Beauducel and Wittmann, 2005).

**Table 4:** Indices of model fit measurement.

**Variable**	**Goodness of fit indexes**						
	**χ^2^**	**Df**	***P***	**CFI**	**RNI**	**SRMR**	**RMSEA**
Model 1: first order factor	663.231	152	<0.001	0.905^*^	0.905^*^	0.106	0.103
Model 2: second-order factor	402.303	147	<0.001	0.953^*^	0.953^*^	0.086^*^	0.074^*^

CFI, robust comparative fit index (^*^cut-off value 0.9); RNI, Relative Noncentrality Index (^*^cut-off value 0.9); SRMR, robust standardized root-mean-square residual (^*^cut-off value 0.08); RMSEA, robust root mean square of approximation (^*^cut-off value 0.06).

### Reliability

After translating the instrument into Indonesian and conducting a data collection, the reliability of the original instrument was analyzed using the McDonald's Omega (ω). The work value items showed that the point estimate is 0.827, which had a good reliability (95% CI: 0.800–0.855). This indicates that the adapted items are well-represented work value in the Indonesian context.

#### Evidence Based on Relation to Other Variable

We used evidence based on relation to other variables to calculate the third evidence of validity. We calculate the Pearson's correlation coefficient between work value and each dimension to career development learning. In simple terms, the direction of the correlations is consistent with what was expected (see Table 5). Work value has a stronger and more significant relationship on the total score compared to the dimension. The most strongly correlated work value dimension is the extrinsic reward (0.683), while the lowest correlated work value dimension is leisure (0.424).

**Table 5:** Mean, SD, and correlation matrix.

**Variable**	**M**	**SD**	**1**	**1a**	**1b**	**1c**	**1d**	**1e**	**2**
1. Work value	4.18	0.412	1						
1a Leisure	3.86	0.721	0.658^*^	1					
1b Intrinsic reward	4.34	0.789	0.815^*^	0.362^*^	1				
1c Altruistic reward	4.51	0.822	0.593^*^	0.209^*^	0.400^*^	1			
1d Social reward	4.35	0.748	0.622^*^	0.145^*^	0.492^*^	0.471^*^	1		
1e Extrinsic reward	3.92	0.807	0.728^*^	0.348^*^	0.381^*^	0.371^*^	0.382^*^	1	
2. Career development learning	4.14	0.530	0.833^*^	0.424^*^	0.604^*^	0.632^*^	0.678^*^	0.683	1

*p < 0.05*,

* *p < 0.01*.

## Discussion

In this study, we have adapted a valid and reliable scale to measure work value for final year university students, specifically in the Indonesian context. The psychometric properties and evaluation of the instrument suggested adequate performance that presents similar psychometric features to the original version (Twenge et al., 2010). Coefficient reliability at or above 0.7 was considered acceptable (Lance et al., 2006). Overall total McDonald's Omega coefficient was satisfactory (0.827). However, we cannot calculate the reliability coefficient for each dimension of work value since there were two dimensions (social reward and altruistic reward) with only two items. Hence, it could be a consideration in the number of items developed for future study.

According to the result from CVI, the first step of the validation process, the expert stated that all the items are relevant to the concept of work value. CVI value is 1.0, which means that this scale has an appropriate sample of items to represent the construct, i.e., whether the items adequately represent the domain of content for the construct. The instrument is appropriate for motivational value mapping. All the items have successfully elaborated in the context of Indonesia's final year student. For example, in the original instrument, the first item in leisure written “a job where you have more than 2 weeks' vacation.” The phrase “2 weeks vacation” is not relevant to Indonesia's general work holiday regulation. The item then changed and emphasized the word “holiday” without considering its “2 weeks” period.

The second validation stage is based on the internal structure using confirmatory factor analysis. We compared the two models, i.e., first order and second order and the second order was the best fit model. It represents that work value consists of leisure, intrinsic reward, altruistic reward, social reward, and extrinsic reward. Each dimension consisted of 2–7 observed variables (item) and had a good loading factor. It also becomes clear that motivational value can be represented by the all the five dimensions and items to describe the work preferences for Z-generations. All the dimensions correlated significantly to the total score of work value. This is a promising instrument of work value suits for Generation Z as an alternative for the previously (Schwartz and Boehnke, 2004; Krumm et al., 2013; Ros et al., 2015; Lechner et al., 2017).

The third validation stage is evidence based on relation to other variable. Some previous study found that work value orientation plays an important role in various career consequences such as career planning, career decision-making, career self-efficacy, career sustainability, career maturity, and career certainty (Chi et al., 2019; Doo and Park, 2019; Kelly et al., 2020). Based on the result of this study, we found that work value significantly correlated with career development learning (CDL). CDL is a process of students learning for their career and getting their self-awareness in terms of interests, values, motivations, and abilities (Pool et al., 2014). CDL will equip them to decide what type of occupation they would find satisfying. The high level of CDL can describe the clear and strong motivational work value. Work value presents cognitive expressions of the needs or goals that bring meaning to the student's future workplace (Pryce, 2016). They are a specific subset of general life values and are influenced by internal and external factors, including their effort to understand and be aware of themselves.

The Pearson's correlation coefficient on each dimension of work value reflects that extrinsic reward shows a stronger correlation with career development learning (CDL). Extrinsic work values refer to instrumental rewards of a job to the work itself, such as a good salary and job security. Jin and Rounds (2012) stated that extrinsic value would increase in young adulthood (22–26 years), the age range of final year students in higher education. The social and intrinsic rewards also have a strong correlation and more significant factor loading. From a relational lens, it is also known that social relationships play a central role in an employee's decision to stay or leave or choose a job in the organization (Jo and Ellingson, 2019). Today's final year student feels a constant need for social connection (Twenge et al., 2010). The intrinsic value gives the specific meaning for students to improve their ability, allow learning the various skills, sharpen the specific skill, and make a personal meaning for the work. The intrinsic reward is core criteria for protean students (Abessolo et al., 2017). They will take action to achieve success in their future career and one's motivation to adapt to a changing environment (Gubler et al., 2014). The students will value autonomy and seek challenges, creativity, and affiliation across varied professional development.

All the evidence for the validity of the questionnaire was encouraging. The instrument may impact future study, practice, and society. Items of the five dimensions (leisure, extrinsic rewards, intrinsic rewards, altruistic rewards, and social rewards) have good reliability. It is used a 5-point Likert-type scale ranging from 1 = very unimportant, 2 = unimportant, 3 = undecided, 4 = important, and 5 = very important. This study has contributed to the study on work value in today's generation and might be used for last year's students in Indonesia to assess themselves before considering job choice after graduation. This instrument properly measures students' job preferences that have a specific value in their future work. Practically, it will be an important instrument for career centers in university to help students to setup career planning for their future work.

Work value instrument validation clearly highlights the need for a context-specific tool to measure the work value since all the dimensions herein are related to the specific condition of future work of final year students, specifically in Indonesia. It is also argued that value could be a motivating factor for fresh graduates, their work priorities, and their determination for future job seeking (Lofquist and Dawis, 1978; Shujaat, 2014). Therefore, it is time to assess the work value for career consideration of final year students and take into account the context of a motivational factor of the students. Several studies agree that the congruence between work value profiles will be associated to positive outcomes in college such as persistence and academic achievement (Balsamo et al., 2013). Then, it helps the student to explore their career development optimally.

However, the generalization of the instrument should be taken cautiously, given that the sample is from one university only. The external validity can be expanded by obtaining the data from universities in Indonesia. In addition, the chi-square value obtained does not meet sufficient criteria. We suggest that further studies involve a more extensive population for the respondents. Since two values only have two observed variables (items), we suggest that further study give some additional items to meet the rule of psychometric calculation and analysis.

## Data Availability Statement

The datasets presented in this study can be found in online repositories. The names of the repository/repositories and accession number(s) can be found in the article/supplementary material.

## Ethics Statement

Ethical review and approval was not required for the study on human participants in accordance with the local legislation and institutional requirements. The patients/participants provided their written informed consent to participate in this study.

## Author Contributions

All authors contributed to the study conception, design and written, and checked the manuscript.

## Conflict of Interest

The authors declare that the research was conducted in the absence of any commercial or financial relationships that could be construed as a potential conflict of interest.

## Publisher's Note

All claims expressed in this article are solely those of the authors and do not necessarily represent those of their affiliated organizations, or those of the publisher, the editors and the reviewers. Any product that may be evaluated in this article, or claim that may be made by its manufacturer, is not guaranteed or endorsed by the publisher.
